# Occurrence of Bovine Cysticercosis in Two Regions of the State of Tocantins-Brazil and the Importance of Pathogen Identification

**DOI:** 10.3390/pathogens8020066

**Published:** 2019-05-20

**Authors:** Benta Natânia Silva FIGUEIREDO, Ricardo Alencar LIBóRIO, Megumi SATO, Camila Figueira da SILVA, Ronaldo Alves PEREIRA-JUNIOR, Yuichi CHIGUSA, Satoru KAWAI, Marcello Otake SATO

**Affiliations:** 1 Escola de Medicina Veterinária e Zootecnia, Universidade Federal do Tocantins, Araguaína 77804-970, Tocantins, Brazil; benta_naty@hotmail.com (B.N.S.F.); camila21_fi@hotmail.com (C.F.d.S.); ronaldo_pgtu@hotmail.com (R.A.P.-J.); 2 Instituto Federal do Tocantins, Gurupi 77410-470, Tocantins, Brazil; ricardolib_63@hotmail.com; 3 Graduate School of Health Sciences, Niigata University, Niigata 951-8518, Niigata, Japan; satomeg@clg.niigata-u.ac.jp; 4 Centro Universitário de Goiás, Uni-Anhanguera, Goiânia 74423-115, Goiás, Brazil; 5 Department of Tropical Medicine and Parasitology, Dokkyo Medical University, Mibu 321-0293, Tochigi, Japan; ychigusa@dokkyomed.ac.jp (Y.C.); skawai@dokkyomed.ac.jp (S.K.)

**Keywords:** public health, *Cysticercus bovis*, *Taenia saginata*, Tocantins, food security

## Abstract

Bovine cysticercosis, caused by *Taenia saginata* metacestodes, is the cause of significant economic losses to the meat production chain by condemnation and downgrading of infected carcasses. It is also a public health issue causing human taeniasis. This study evaluated the occurrence of bovine cysticercosis at the meat inspection procedures in slaughterhouses of south and north regions of the Tocantins State in Brazil. Specimens identified as cysts of *T. saginata* were collected and analyzed by molecular (PCR) and histopathological techniques. The cysts were collected from March to December of 2010 in slaughterhouses located in the cities of Alvorada (South) and Araguaína (North). The frequency of cystic lesions during the study was 0.033% (53/164,091) with 69.81% of calcified lesions and 30.9% of live cysts at meat inspection. From 14 samples submitted to molecular analysis, 28.57% (4/14) were positive for *T. saginata*. The histopathological analysis of the non-*T. saginata* samples showed lesions suggestive of granuloma and hydatid disease. The results indicated that the identification of the etiological agent is difficult by macroscopic inspection, emphasizing the need to associate specific diagnostic methods at meat inspection in abattoirs. In addition, species-specific PCR would be an effective tool for diagnosis, monitoring, and identifying cysticercosis, assisting the conventional tests.

## 1. Introduction

**Figure 1:**
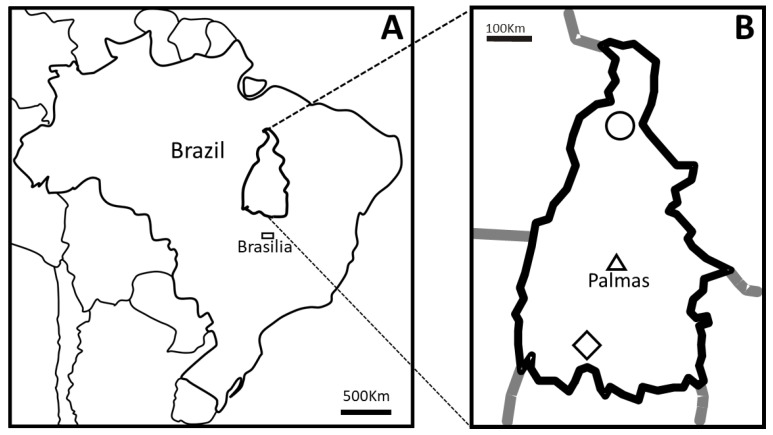
Map of Brazil indicating the position of the State of Tocantins (**A**) and the Country’s capital city Brasília (rectangle). The material used in this study were collected in slaughterhouses in the municipalities of Araguaína (circle) in the north and Alvorada (diamond) in the south of Tocantins (**B**). The triangle in B indicates Palmas city, the capital of the State of Tocantins.

Brazil has the second largest cattle herd in the world with about 209 million heads, the second largest producer and exporter of beef [1]. The state of Tocantins, located in the eastern Amazon region of Brazil (Figure 1A), has a cattle herd of 6.3 million heads, bred by extensive farming in wide areas of savanna, fed mainly in natural grass spread across all the regions of the state [2]. The quality of the meat produced in the Tocantins made the state a main producer of beef for Brazilian and international markets, reaching more than 20 Countries in Europe and Asia [3].

Bovine cysticercosis caused by *Taenia saginata* is an important cattle disease, causing economic losses to the meat production chain due to the condemnation and downgrading of infected carcasses [4]. Furthermore, this cestode causing taeniasis in humans is a health issue, especially in regions with poor sanitary conditions, associated with socioeconomic and cultural aspects as the consumption of undercooked or raw beef [5,6]. Humans are infected by ingestion of viable *T. saginata* cysts in meat, the adult worm develops in the small intestine and releases gravid proglottids full of eggs in the feces contaminating the environment in areas with no sewage treatment. Bovines are infected by ingestion of grass with eggs developing cysts in striated muscles, also called *Cysticercus bovis* [7].

Routinely, the diagnosis is made by macroscopic examination during the post-mortem inspection of carcasses; however, the method has been criticized by low sensitivity in detecting [8] and limited diagnostic capability [9]. On the other hand, molecular techniques such as PCR have high sensitivity and specificity, allowing effective identification and differentiation of species of *Taenia*, overcoming many disadvantages of conventional methods [10,11,12]. There are few studies on bovine cysticercosis in the state of Tocantins with different prevalence estimations varying from 0.02% to 10.23% [13,14]

In this sense, this study aimed to verify the occurrence of bovine cysticercosis in slaughterhouses and confirm how molecular identification could better identify cases of the disease in meat inspection, using cysts collected in the south and north regions of the State of Tocantins (Brazil) (Figure 1B).

## 2. Results

**Figure 2:**
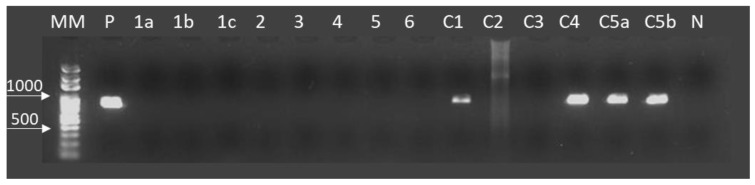
Amplification of *CO1* by *Taenia saginata* specific PCR in DNA purified from cystic lesions of bovines in Araguaina and Alvorada, Tocantins, Brazil. From left to right are; DNA size markers (MM), *Taenia saginata* DNA (accession no. AB107246) as a positive control (P), tested samples from 1a to c5b and bovine genomic DNA as a negative control (N). The cystic samples 1a, 1b, 1c, 2, 3, 4, 5, 6, C2, and C3 were not detectable using PCR in this study. The cysts C1, C4, C5a, and C5b were identified as *T. saginata* presenting an amplicon of 827 bp. C1 was the only calcified cyst presenting amplification by PCR.

In Table 1, the monthly number of bovines processed in each facility and the number of cases of viable and calcified cysticercosis referring to the period is shown. The total frequency of the bovine cysticercosis during the collection period (from March to December 2010) was 0.033% (53/164,091) with 30.19% (16/53) of live cysts and 69.81% (37/53) of calcified cysts. The prevalence of the bovine cysticercosis was 0.037% in Alvorada, with 0.015% live cysts (09 cases), and 0.022% calcified cysts (13 cases). The prevalence of live and calcified cysts was 0.006% (7 cases) and 0.023% (24 cases), respectively, in Araguaína, with a prevalence of 0.029% (Table 1). Based on the PCR technique, 28.57% (4/14) of collected cysts were identified as *T. saginata* (Figure 2, Table 2). In the present work, 90% (9/10) of the calcified cysts found at meat inspection were not detected by specific PCR.

**Figure 3:**
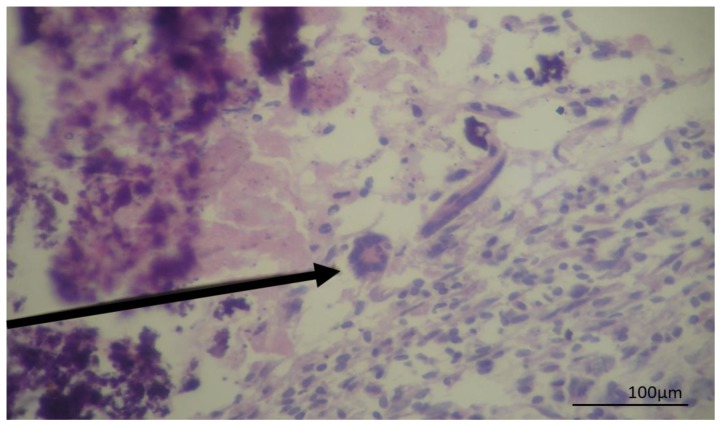
Photomicrography of a cyst recovered from a bovine liver showing a granulomatous lesion in hematoxylin and eosin stain. The arrow indicates Langhan’s giant cells with broad cytoplasm and nuclei arranged at the periphery of the horseshoe-shaped cell.

**Figure 4:**
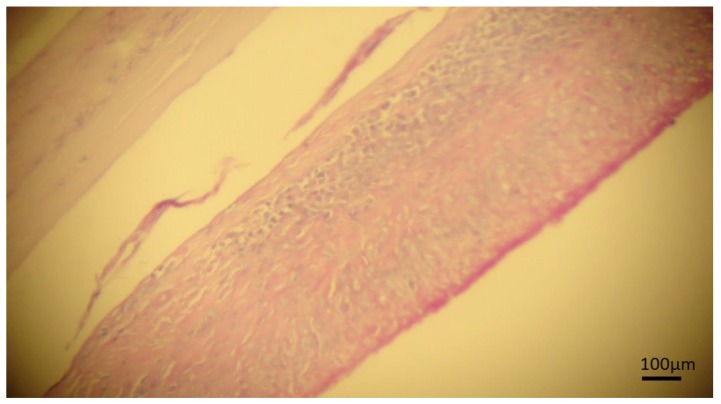
Photomicrography of cyst in bovine liver stained by periodic acid–Schiff stain (PAS). Continuous laminated membrane and fibrous capsule, a strong granulomatous reaction with the presence of germinative tissue is observed suggesting hydatidosis.

The microscopic analysis of negative samples in the PCR for cysticercosis revealed that three samples showed lesions with characteristics of granuloma were observed multinuclear cells in a granulomatous lesion (Figure 3). In addition, three different cysts sections stained by PAS, showed laminated membranes and germinal layers in magenta color, a characteristic of hydatid cysts (Figure 4). Four samples presented inconclusive results.

## 3. Discussion

The results found in this study showed a low frequency of bovine cysticercosis when compared with the data described by Marques et al. (2008) in a study with prevalence data in several Brazilian states, reporting a prevalence of 10.23 % of bovine cysticercosis in the State of Tocantins, which is higher than other Brazilian states as São Paulo (8.76%), Paraná (7.53%), Minas Gerais (5.92%), Mato Grosso do Sul (4.74%), Goiás (4.16%), and Mato Grosso (0.71%) [13]. However, the official data of the prevalence of cysticercosis cases in the state of the Tocantins in 2009 and 2010 was 0.02% [14], near our findings of 0.033% total prevalence. There was no targeted action directed for taeniasis/cysticercosis control in the state of Tocantins, and the sharp decrease of bovine cysticercosis prevalence in 10 years could be related with the improvements in education, health infrastructure built after the creation of the state in 1989 and the urbanization of the population from 40.1% in 1980 to 78.8% in 2010 [15].

The macroscopic examination of mineralized (calcified) cysts, generally classifying it as calcified cysticercosis may overestimate the results of the prevalence of the bovine cysticercosis, as a specific diagnosis of calcified cysts is very difficult by post-mortem inspection [16,17,18].

It is not possible to discard the possibility of cysticercosis indeed; however there is a higher possibility of false-positive results by eye inspection once macroscopically, calcified hepatic nodular lesions are difficult to diagnose due to intense inflammatory reaction [16]. In this aspect, the histopathological examination may help in the identification of the causative agent, with the visualization of microscopic characteristic structures, as a laminated membrane in hydatidosis and calcareous corpuscles in cysticercosis [16,18,19,20]. In calcified cysts, DNA can be detected (with a lower sensitivity) by PCR [19]. PCR could not detect most *T. saginata* in the calcified cysts in this study, probably because of the decrease in sensitivity by DNA degradation in old cysts.

The definitive diagnosis of hydatidosis is usually based on histopathological analysis using PAS staining, where the laminated membrane, germinal layers, and protoscolices are PAS-positive staining in magenta color [20,21], the membrane staining characteristics were observed in three cysts in the present study, however, protoscolices were not observed (Figure 4).

The presence of granuloma with multinucleated giant cells indicates chronic inflammation. In those granulomatous lesions, there is focal necrosis with mineralization, the presence of macrophages, epithelioid cells, and Langerhans cells resulting from the fusion of epithelioid cells, there are different origins for this type of pathological lesion including mycobacterium and other bacterial infections, fungi, and protozoa [22,23] but not cysticercosis. In our histopathological evaluation, despite not all characteristics of granuloma being observed, it is possible to detect multinucleated cells, suggesting the cystic lesion could be or evolve in a granuloma.

As demonstrated in the present study, the visual inspection may present limitations in the judgment of cysts, mainly those calcified, since these have similar macroscopic characteristics with various agents, this may cause distortions in data of occurrence and prevalence reports.

*T. saginata* was found in the two regions studied, in the North and South areas of the State of Tocantins. Although the low prevalence of bovine cysticercosis, the absolute numbers are relevant, indicating environmental contamination and the need for basic sanitary care. Twenty-two infected animals in the south region and 31 in the north region presented cysticercosis. These results indicate the presence of human taeniasis and the contamination of the environment with parasite eggs is probably due to the lack of sanitary education and inadequate waste treatment [24,25,26].

This study highlights the importance of associated diagnostic methods for improved diagnosis in meat inspection with molecular detection as an accurate alternative for monitoring data, which may be overestimated by conventional techniques.

## 4. Materials and Methods

### 4.1. Collection of Cysts

To evaluate the specificity of gross examination, fourteen samples of cystic lesions of cattle were collected during the post-mortem inspection in the slaughterhouses of Alvorada and Araguaína, which are the main processors of livestock from cities located in the south and north regions of the State of Tocantins (Brazil), respectively, from March to September of 2010 (Figure 1).

The post-mortem examination of the carcasses followed the routine protocols to cysticercosis investigation in the inspection line in abattoirs and constituted by the slicing and inspection of the muscles of head, tongue, heart, diaphragm, and esophagus, according to the regulations of the Department of Inspection of Animal Origin Products (DIPOA) [27].

The cysts were classified in live or calcified by morphologic characteristics at the inspection line and were dissected, packed, labeled, and stored at 4 °C before sending to the laboratory (Laboratório de Parasitologia Veterinária) of the Universidade Federal do Tocantins for further analysis.

### 4.2. DNA Extraction and PCR

Cysts were individually cut in small pieces, transferred to microtubes, and disrupted using a pestle. The tubes were incubated at 55 °C in lysis buffer (100mM Tris HCl pH 8.5, 0.5M EDTA, 10% SDS, 5M NaCl, 20mg/mL Proteinase K) until complete digestion. Genomic DNA of each cyst was extracted and purified using the phenol/chloroform/isoamyl alcohol method and purified by ethanol precipitation [28]. The identification of *T. saginata* was carried out using the species-specific PCR targeting the mitochondrial *CO1* with an amplicon of 827 bp as described previously [11] with some modification as follows: instead of multiplex PCR, the PCR was done using the specific forward primers to *T. saginata* (H05 TsagF) and the reverse primer (E03 RevCOI). PCR reactions were done using KOD Plus Neo (Toyobo, Japan), amplifications were conducted in 50 µl total volume using 1 µl of template DNA and 12.5 picomoles of each primer. The PCR conditions were: 1 min to 98 °C followed by 35 cycles of 94 °C 60 s, 58 °C 30 s, 72 °C 60 s and a final extension of 72 °C for 5 min. Controls of PCR were added in each run. *T. saginata* with Brazilian origin (AB107246) genomic DNA (1 ng) as a positive control [11] and bovine genomic DNA from our DNA panel (1 ng) as a negative control of the reactions. The amplicons were visualized in 1.5% agarose gels under UV light by ethidium bromide staining.

### 4.3. Histopathological Evaluation

Part of the cyst wall together with the adjacent tissue of liver and muscle were collected and fixed in 10% buffered formalin for histopathological studies. Tissue samples were prepared in paraffin and sections 5 μm thick were stained using hematoxylin and eosin (HE) and periodic acid-Schiff stain [29].

## Figures and Tables

**Table 1:** Total number of heads processed in the slaughterhouses of this study and the cases of viable (VI) and calcified (CA) cysticercosis diagnosed between March and September of 2010 by the inspection service.

Month	Alvorada	VI	CA	Araguaina	VI	CA
March	8564	01	01	15,788	03	01
April	8811	01	02	14,863	-	04
May	8772	02	-	13,379	-	01
June	9270	01	01	16,050	-	06
July	7731	02	04	18,428	02	07
August	7998	01	03	13,754	01	03
September	7445	01	02	13,238	01	02
**Total**	**58,591**	**09**	**13**	**105,500**	**07**	**24**

**Table 2:** Cyst samples collected for molecular analysis (PCR) from March to September 2010 in Alvorada city (A), Southern area, and Araguaina city (B) in the Northern area of the State of Tocantins, Brazil. The cysts were classified in viable (VI) and calcified (CA) and by the localization of the cyst.

Sample ID	Classification	Slaughterhouse	Collection Date	Local	PCR
C1	CA	B	March	Heart	P
C2	CA	B	April	Heart	N
C3	CA	B	April	Heart	N
C4	VI	A	April	Masseter	P
C5a	VI	A	July	Masseter	P
C5b	VI	A	July	Masseter	P
1a	CA	B	June	Masseter	N
1b	CA	B	June	Masseter	N
1c	CA	B	June	Masseter	N
2	VI	B	August	Heart	N
3	CA	A	August	Heart	N
4	CA	A	August	Masseter	N
5	CA	A	August	Masseter	N
6	CA	A	September	Liver	N

P–positive; N–negative.
